# Visual evoked potential evaluation following brain injury in school-aged children

**DOI:** 10.22336/rjo.2024.05

**Published:** 2024

**Authors:** Victoria Verejan

**Affiliations:** * Department of Ophthalmology, “N. Testemițanu” State University of Medicine and Pharmacy, Chişinău, Republic of Moldova

**Keywords:** visual evoked potentials, brain injury, children

## Abstract

**Aim:** The research aimed to establish reference values of visual evoked potentials among school-aged children after brain injury.

**Methods:** Eighteen patients with persisting visual symptoms after brain injury have been examined. A pattern-VEP test has been used during the examination.

**Results:** The prolongation of the N2 wave in 55,6%-66,6%, P wave in 55,7%-66,7%, and N3 wave in 16,7%-22,2% was determined in the research group. Likewise, the decrease in the amplitude of the P wave was determined in the case of 16,7%-33,3%. According to the topography, we concluded that the prechiasmatic alteration was predominantly determined as bilateral in the optic pathways, with emphasis equally on the right and left.

**Conclusions:** VEP evaluation remains one of the most credible methods of examination. In the case of moderate or severe traumatic optic neuropathy, it allows the detection of damage to the optic pathways before the appearance of organic changes that are often irreversible. The possibility of early detection of such modifications could justify the initiation of a dosed stimulatory treatment, to avoid damage to the optic pathways that would induce secondary optic atrophy.

**Abbreviations:** VEP = visual evoked potentials, MRI = magnetic resonance imaging

## Introduction

Visual evoked potentials represent an exploration with high sensitivity that refers to the electrophysiological signal arising from the neural activity correlated to the corresponding region of the visual cortex in response to a certain visual stimulus fixed in time [**1**,**2**]. The primary recorded activity refers to the activation of the photoreceptor cones in the central area at 150 of the visual field [**1**-**4**]. This corresponds to ~50% of the primary visual cortex [**4**,**5**]. The recording of VEP activity has been established as a method that provides quantitative data that describes the integrity of the primary visual pathways [**6**-**9**]. When applying a stimulus of high luminance and low temporal frequency, parvocellular pathways dominate in visual information processing [**10**], and on the other hand, when using a visual stimulus of low luminance and high temporal frequency, magnocellular pathways dominate. Recent studies confirm that patients with a history of cerebral injury have a slow ability to track a fixed target. This fact suggests damage to the magnocellular pathways [**11**]. Thus, we could conclude that subjects with a history of cerebral injury present a disturbance of the magnocellular pathways [**12**-**14**], which, in turn, are responsible for the processing of visual information with low luminance. In their case, we will observe VEP data with lower amplitude than in control subjects [**15**].

The VEP test represents a graphic recording of electrical activity in the occipital cortex by stimulating the visual receptors. The stimuli used are of two types: bright flash (white light) and stimulus pattern, structured in a checkerboard, with good black-white contrast. If changes are determined along the optic pathways, the VEP examination will show changes in the latency, amplitude, and shape of the resulting waves, showing disturbances of certain peripheral portions of the optic pathways (retinal, optic nerve lesions). VEP recording can detect lesions in the early stages, when they are not clinically detectable and this is an important means of diagnosis. The patient is exposed to a series of flash-type visual stimuli in front of a monitor that would have the appearance of a checkerboard that includes black and white squares. The color changes alternately with a preset frequency. Electrodes are applied according to an international scale that records the cerebral activity arising from the visual stimulation, with the subsequent graphs ‘generation. Each patient is asked to sit as comfortably as possible at approximately 1 m from the monitor, without making additional movements. Blinking is allowed but without exaggeration. A red landmark appears in the middle of the checkerboard to help patients fixate their gaze and accommodate to maintain visual attention. In turn, the patients are informed to fix the landmark without making excessive movements [**1**]. The VEP-Flash test is applied to patients with very low acuity or uncooperative patients so that the latency of the recorded wave (P) shows the state of the optic nerve. The criteria for a VEP response to a light stimulus within normal limits would be the appearance of a positive wave at a latency of approximately 100 ms, used as reference values. Three components of this wave can be distinguished: the initial one being negative (N2) recorded as having a latency of 70, the second component being a positive one (P) with a latency of 100 ms, and the third equally negative (N3) with 155 ms latency. P2 wave values are considered more objective in a VEP examination because they show minimal variability among individuals.

The following terms will be used in the context as defining notions: the latency of the wave that represents the time required for the initial visual stimulus to reach the cortical response measured in ms, the amplitude of the wave that describes the height of the P wave and represents the quantitative assessment of the generated visual stimulus perceived by the cortex. The P wave is generated in the striate and peristriate occipital cortex due to the activation of the primary visual cortex, also following the discharge of thalamocortical fibers [**15**].

At the age of 3, the child can cooperate toward the VEP assessment. The problem with such an investigation is not the immaturity of the visual system, but the inability to maintain fixation for a predetermined time. The development of the visual system, the degree of myelination as well as the parameters of visual acuity makes it possible to generate adult-like VEP waves already at the age of 6 years. Certain deviations that may appear during the adolescent period are determined, but these are non-essential. The amplitude of the P wave becomes maximal at the age of 7-8 years, and the brain reaches the dimensions of an adult at the age of 6. In the preadolescent period, the size of the brain is maximal regarding the cranial skeleton and facial muscles. As the child matures, the density of these tissues increases so that both the amplitude and the latency of the P wave decrease. These parameters remain stable until approximately 28 years of age, after which they begin to decrease in value [**16**]. The placement of the electrodes is predetermined, being connected to the “10-20 International System”. The middle occipital electrode (0Z) is placed on the midline, above the inion (occipital protuberance) approximately 3-4 cm for adults. The lateral electrodes (01, 02) are positioned at equal distances from the median.

Most of the primary visual cortex is found in the sulci and not on the cortical surface of the occipital lobe. With precision, we can state that only 10 concentric degrees of the visual field find their projection on the cortical surface of the occipital lobe. Moreover, the structure of the cortex of the occipital lobe is so different not only from individual to individual but even from hemisphere to hemisphere. According to research studies of the firing of visual potentials visualized using contrast MRI, it has been shown that the cortical surface is responsible for the firing of the N1 wave. The area responsible for generating the P wave is the extrastriate cortex of the middle occipital gyrus. The third N2 wave is generated from several areas, even having as its source an area located deep in the parietal lobe [**17**].

Concerning the pediatric population, few studies determine reference values. Somehow, benchmarks for N2 wave latency were determined within the 6 to 20-year age group of 63.84±3.5 for the right eye and 64.64±4.85 for the left eye, respectively. The N3 wave latency values were determined to be 154.15±5.13 for the right eye and 152.76±5.13 for the left eye, respectively. For the P wave latency, the determined values were 96.7±7.31 for the right eye and 96.52±9.68 for the left eye, respectively. The amplitude of the P wave was evaluated to be 14.4±2.42 for the right eye and 16.9±4.16 for the left eye respectively [**15**].

## Materials and methods

A total of 18 patients were selected as the research group (L1), without post-surgical sutures applied to the scalp and excoriations present in the occipital region, and from the control group. Another 18 patients have been selected as the control group (L0), children having no organic visual disturbances and other sorts of chronic diseases. 

**Fig. 1:**
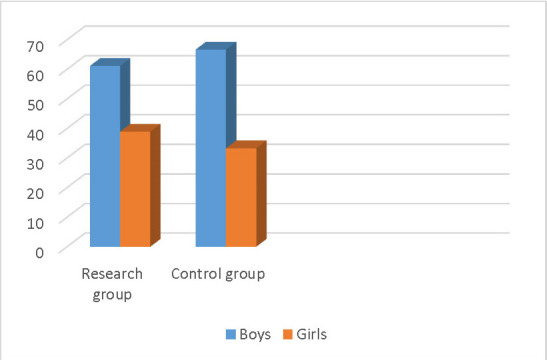
Patients repartition in groups by sex

The age of the children was between 10-18 years, and the distribution by gender was as follows: for the researched group: boys constituted 61,1% and girls 38,9%, and in the control group 66,7% boys and 33,3% girls were included (**Fig. 1**).

The values of the latency of the N2 wave in the patients of the research group, for the right eye, were determined within the range of 60-70 ms in 8 patients, constituting 44,4%, another 8 patients recorded maximum values in the range of 71-80 ms, respectively 44,4% and 2 patients reported maximum values of 80 ms and more (11,2%). The data concerning the control group were: values in the range of 60-70 ms in 11 patients, constituting 61,1%, another 7 patients recorded maximum values in the range of 71-80 ms, respectively 38,9%. The values of the latency of the N2 wave in the patients of the research group, for the left eye, were determined within the range of 60-70 ms in 6 patients, constituting 33,4%, another 11 patients recorded maximum values in the range of 71-80 ms, respectively 61,1% and 1 patient reported values of 80 ms and more. The data regarding the control group were: values in the range of 60-70 ms in 15 patients, constituting 83,3%, and another 3 patients recorded maximum values in the range of 71-80 ms, respectively 16,7% (**Table 1**).

**Table 1:** Values of the N2 wave latency

Eye	Values	L1		L0	
		Patients	%	Patients	%
Right	60-70 ms	8	44,4	11	61,1
	71-80 ms	8	44,4	7	38,9
	>81-90 ms	2	11,2	0	0
x2=2,540a, gl=2, p1=0,281					
Left	60-70 ms	6	33,4	15	83,3
	71-80 ms	11	61,1	3	16,7
	>81-90 ms	1	5,5	0	0
x2=9,429a, gl=2, p1=0,009					

The P wave latency values in patients from the research group, for the right eye, were determined within the range of 90-120 ms in 4 patients, constituting 22,2%. On the other hand, most patients presented values higher than the reference range in the number of 10, respectively 55,7%, and 4 patients reported values lower than the reference interval (22,2%). The values for the control group were as follows: the 90-120 ms interval was present in 16 patients, constituting 88,9%, and in the case of 2 patients (11,1%), higher values than the reference interval were determined. The P wave latency values in the patients of the research group, for the left eye, were in the range of 90-120 ms in 5 patients, constituting 27,8%. Most of the patients presented values higher than this interval - 12 patients (66,7%), and in 1 patient (5,5%) a lower value than the reference interval was determined. The values for the control group were as follows: the 90-120 ms interval was present in 16 patients, constituting 88,9%, and in the case of 2 patients (11,1%), higher values than the reference interval were determined (**Table 2**).

**Table 2:** Values of the P wave latency

Eye	Values	L1		L0	
		Patients	%	Patients	%
Right	90-120 ms	4	22,2	16	88,9
	> 90-120 ms	10	55,6	2	11,1
	< 90-120 ms	4	22,2	0	0
x2=16,533a, gl=2, p1=0,001					
Left	90-120 ms	5	27,8	16	88,9
	> 90-120 ms	12	66,7	2	11,1
	< 90-120 ms	1	5,5	0	0
x2=13,829a, gl=1, p1=0,001					

The values of the latency of the N3 wave in the patients of the research group, for the right eye, were determined within the range of up to 150 ms in 9 patients, constituting 50%, another 6 patients recorded values in the range of 151-159 ms, respectively 33,3%, as well as 3 other patients (16,7%) who reported values of 160 ms and more. For the control group, the following distribution was determined: the interval up to 150 ms - 10 patients, constituting 55,6%, and in 8 patients (44,4%) values included in the interval 151-159 ms. The values of the latency of the N3 wave in the patients in the research group, for the left eye, were determined within the range of up to 150 ms in 9 patients, constituting 50%, another 5 patients recorded values in the range of 151-159 ms, respectively 27,8%, as well as 4 other patients (22,2%) who reported values of 160 ms and more. For the control group, the following distribution was determined: the range up to 150 ms - 12 patients, constituting 66,7%, and in 6 patients (33,3%) values included in the range of 151-159 ms (**Table 3**).

**Table 3:** Values of the N3 wave latency

Eye	Values	L1		L0	
		Patients	%	Patients	%
Right	Until 150 ms	9	50	10	55,6
	151-159 ms	6	33,3	8	44,4
	> 160 ms	3	16,7	0	0
x2=3,600a, gl=2, p1=0,165					
Left	Until 150 ms	9	50	12	66,7
	151-159 ms	5	27,8	6	33,3
	> 160 ms	4	22,2	0	0
x2=4,519a, gl=2, p1=0,104					

The amplitude of the P wave was determined in the patients of the research group, for the right eye, with values up to 10 µV in the case of 6 patients, corresponding to 33,3%, another 6 patients expressed values between 11-20 µV, 33,3%, and 6 children showed values of 21 µV and more (33,3%). For the control group, the following distribution was determined: the range of up to 10 µV in the case of 4 patients, corresponding to 22,2%, 1 patient expressed values between 11-20 µV, 5,5%, and 13 children showed values of 21 µV and more (72,3%). The amplitude of the P wave for the left eye in patients from the research group had values of up to 10 µV in the case of 3 patients, corresponding to 16,7%, another 9 patients expressed values between 11-20 µV, 50% and 6 showed values of 21 µV and more (33,3%). The following distribution was determined for the control group: the range of 11-20 µV in the case of 4 patients, corresponding to 22,2%, and the other 14 patients (77,8%) expressed values of 21 µV or more (**Table 4**).

**Table 4:** Values of the P wave amplitude

Eye	Values	L1		L0	
		Patients	%	Patients	%
Right	Until 10 µV	6	33,3	4	22,2
	11-20 µV	6	33,3	1	5,5
	21µV and more	6	33,3	13	72,3
x2=6,550a, gl=2, p1=0,038					
Left	Until 10 µV	3	16,7	0	0
	11-20 µV	9	50	4	22,2
	21µV and more	6	33,3	14	77,8
x2=10,186a, gl=2, p1=0,006					

**Fig. 2:**
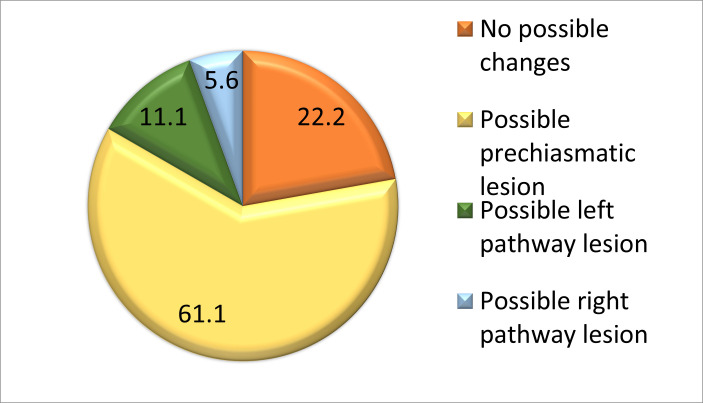
Possible visual pathway disturbance in school-aged children after brain injury according to VEP recording

According to the topography of the condition, the examined patients presented the following distribution. In the case of 4 patients (22,2%), no possible diseases of the pathways were determined. In the case of 11 patients (61,1%), bilateral prechiasmatic damage to the optic pathways was determined. Two patients (11,1%) presented damage to the optic path on the left side, and 1 patient (5,6%) had damage on the right side (**Fig. 2**).

## Discussions

Examination of visual evoked potentials was determined to be correlated as values of N2, P, and N3 wave latencies within reference ranges. The prolongation of the latencies of the mentioned waves denotes a delayed propagation of the visual stimulus to the cortex. In the research group, the prolongation of the N2 wave was determined in 55,6%-66,6%, of P in 55,7%-66,7%, and respectively of the N3 wave in 16,7%-22,2%. Likewise, the decrease in the amplitude of the P wave was determined in the case of 16,7%-33,3%, which confirmed the damage to the transmission of the visual stimulus in pediatric patients aged 7-18 years after a medium cranio-cerebral trauma. Another distinctive feature is the change in the morphology of the P wave, which also demonstrates the alteration of the transmission of the visual stimulus, the graphic record of which is displayed by the presence of the W wave. According to the topography of the condition, we concluded the predominantly bilateral prechiasmatic alteration of the optic pathways with an equal emphasis on the right or the left.

The evaluation of visually evoked potentials could rightly be considered one of the most sensitive methods aimed at disproving and/or confirming damage to the optic pathways in the acute post-traumatic period. It can elucidate the presence of lesions in doubtful cases and presents an investigation with a high degree of credibility. It is accepted to evaluate the latency and amplitude of N and P waves, which graphically indicate the passage of the visual impulse and its transformation into a nerve stimulus. According to the VEP data, we could conclude that every patient subjected to cranio-cerebral trauma develops a traumatic optic neuropathy anyway, but one of medium severity without the organic affection of the visual analyzer. To confirm this statement, the dysregulation of the latency of the N2 and N3 waves is needed. If we refer to the topography of the condition, bilateral or unilateral prechiasmal damage to the optic pathways will be determined in the case of a third of the patients. However, the VEP evaluation remains one of the most credible, because in cases of moderate or severe traumatic optic neuropathy, it allows detection of damage to the optic pathways before the appearance of organic changes that are often irreversible. The possibility of early detection of such changes could justify the initiation of a dosed stimulatory treatment, possibly steroids, to avoid damage to the optic pathways that would induce secondary optic atrophy.

## Conclusions

1. VEP evaluation showed an alteration of the conductivity of the visual impulse after brain injury in school-aged children, demonstrated by alterations in latency of N2, P, and N3 waves and amplitude of the P wave.

2. The N2 and N3 waves recorded data may not be considered as independent reliable criteria, since the latencies in school-aged children after head injury are within the expected norm values. Referring to our research, this interval would be for the N2 wave of 60-70 ms, and the N3 wave ranged until 150 ms.

3. The latency and amplitude values of the P wave can be considered as a conclusion for a visual impulse alteration. In the case of school-aged children after brain injury, we found a latency delay of more than 90-120 ms and an amplitude decrease of less than 20 µV.

4. Regarding the possible topographical lesion, about 60% of school-aged children show prechiasmatic alteration after brain lesions.

**Conflict of Interest Statement**

The author declares no conflict of interest.

**Informed Consent and Human and Animal Rights Statement**

The informed consent was received from every patient.

**Authorization for the use of human subjects**

Ethical approval: The research related to human use complied with all relevant national regulations and institutional policies, followed the tenets of the Helsinki Declaration, and was approved by the Research Ethics Board of “N. Testemiţanu” State University of Medicine and Pharmacy, Chişinău, Republic of Moldova on 21/05/2018 No. 63. The trial was the authors’ initiative. The author is independent and takes responsibility for the integrity of the data and the accuracy of the data analysis.

**Acknowledgments**

None.

**Sources of Funding**

This study was supported by “N. Testemiţanu” State University of Medicine and Pharmacy, Chişinău, Republic of Moldova. 

**Disclosures**

None.
